# End‐to‐end image analysis pipeline for liquid‐phase electron microscopy

**DOI:** 10.1111/jmi.12889

**Published:** 2020-03-23

**Authors:** G. MARCHELLO, C. DE PACE, A. DURO‐CASTANO, G. BATTAGLIA, L. RUIZ‐PÉREZ

**Affiliations:** ^1^ Physical Chemistry Chemical Physics Division, Department of Chemistry University College London London UK; ^2^ Institute for the Physics of Living Systems University College London London UK; ^3^ The EPSRC/JEOL Centre for Liquid Phase Electron Microscopy at University College London London UK

**Keywords:** Denoising, deblurring, LTEM, membrane

## Abstract

Liquid phase transmission electron microscopy allows the imaging of materials in liquid environments. The sample is encapsulated within electron‐beam transparent windows and hence protected by the ultrahigh vacuum necessary within the electron gun. Such an approach allows to study biological and soft materials in their natural environment and offers the possibility of accessing their dynamic nature. Yet, the electron beam scattering from the windows and solvent increases the image noise and blur. Herein, we propose a pipeline to both de‐noise and sharpen images obtained by liquid transmission electron microscopy. We develop the workflow in a way that it does not require any human interference, nor introduce artefacts, but actually unveils features of the imaged samples covered by the noise and the blur.

**Lay Description:**

Transmission Electron Microscopy TEM is one of the most powerful techniques for structural determination at the nanoscale, with the ability to image matter down to the atomic level. TEM is only possible by keeping the electron beam under high vacuum in order to avoid undesired scattering events in the beam path. High vacuum means that the TEM samples must conventionally be in solid‐state. Thus, samples in liquid form or containing liquids, like water, need special preparation techniques which tend to alter the structure and chemical nature of the sample. Such alterations are particularly critical for biological and soft organic materials where the structures are controlled by the presence of water and/or other liquids. The development of new cameras, materials and sample holders have made possible for TEM to be performed on liquid samples. Liquid Phase Transmission Electron Microscopy (LTEM) offers the possibility to investigate nanoscopic structures in liquid state and monitor dynamic processes. However important limitations come from the liquid nature of samples in the imaging process such as the low contrast afforded by organic and biological materials and additional noise and blur introduced by the liquid sample and its thickness. Existing image analysis algorithms for TEM result inadequate for LTEM. The end‐to‐end image analysis method herein has the ability to recover the original images together with their sharpness, without introducing any artefacts. The proposed algorithms offer the great advantage of unveiling image details which are not usually seen during imaging, thus allowing a better understanding of the nature, structure and ultimately the function of the investigated structures. The fully automatised analysis method allows to efficiently process dozens of images in few hours, improving dramatically the performance of LTEM imaging

## Introduction

Every measurement is comprised of the actual signal and its associated noise. The presence of noise covers and distorts the original signal often modifying structural features of the subject under study. The tremendous advances in imaging techniques achieved in the last decades have in turn generated a vast development in denoising algorithms aimed to restore the signal lying under the noise components. Many existing approaches (Shao *et al*., 2014) have been tailored to each particular imaging process and type of noise (Rohit & Ali, 2013). The specificity associated to denoising algorithms have thus prevented the production of a general high‐performance algorithm that operates in all imaging modalities (Rohit & Ali, 2013). Herein we propose an algorithm that aims to recover images generated by liquid transmission electron microscopy LTEM (De Jonge & Ross, 2011), a cutting‐edge imaging modality that allows real time imaging of nanoscopic structures within liquid and monitor dynamic processes. This approach offers a step‐change in our ability to study matter in its virgin state on the nano and micron scale, removing the artefacts induced by drying or cryogenic treatments. LTEM imaging find applications in a myriad of research fields from electrochemical reactions (Radisic *et al*., 2006), nanocrystals growth (Zheng *et al*., 2009) and tomographic reconstructions of particles in liquid (Marchello *et al*., 2019). However, all these investigations require highly resolved images which need a few postprocessing steps aimed to recover essential features of the structures under study. Conventionally, EM images are heavily affected by different types of noise, mainly due to the fluctuation in the intensity of the beam, secondary electrons and the readout noise of the detector (Narasimha *et al*., 2008). In the case of LTEM the liquid nature of the specimen adds an additional component to the overall existing noise. Hence, as a result existing algorithms are inadequate for restoring LTEM images. The resolution of the images is also further lowered by the presence of the liquid media that adds blurring to the images. In addition, image corruption is remarkably worse when studying organic samples, as they are made of light elements with low electron density. The investigation herein presented aims to improve the resolution of images from organic materials recorded via LTEM.

## Materials and methods

### Materials

#### Polymer synthesis and particle formation

Poly(2‐(methacryloyloxy)ethylphosphorylcho‐line)‐poly(2‐diisopropylaminoethyl methacrylate) PMPC_25_‐PDPA_70_ amphiphilic block copolymers were synthesised by atom transfer radical polymerisation (ATRP) as reported by Du *et al*. (2005). PMPC_25_‐PDPA_70_ polymeric particles were formed by the pH‐switch method. First, the polymers were dissolved in phosphate‐buffered saline PBS at pH 2 in a concentration of 10 mg mL^–1^ solution; then, the pH was slowly raised to 7.4 adding sterile NaOH (0.5 M) dropwise. The initial transparent solution became milky due to the formation of the polymer nanostructures. The solution was purified by size‐exclusion chromatography SEC, where the solution was passed through a size exclusion column filled with Sepharose 4B, bead diameter between 45 and 165 µm, purchased from Sigma‐Aldrich. SEC was performed in order to remove polymer aggregates and larger structures. The PMPC‐PDPA polymer particle solution was diluted to 1.5 mg mL^–1^ in PBS and imaged.

Poly(ethylene glycol)‐poly(methionine) PEG‐PMET Poly(ethylene glycol)‐*b*‐poly(L‐methionine) block copolymer PEG_125_‐PMET_120_ vesicles were produced by the solvent‐switch method. Briefly, PEG_125_‐PMET_120_ (molecular weight (Mw): 25 kDa by ^1^H‐NMR (nuclear magnetic resonance), 26 kDa and 1.4 polydispersity by gel permeation chromatography (GPC)) was previously synthesised according to Yamada *et al*. (2013), with some modifications. 10 mg of the isolated polymer were dissolved in 0.5 mL of a mixture 1 : 1 of tetrahydrofuran: dimethyl sulfoxide (THF:DMSO). Next, 1.15 mL of milliQ water was added using a syringe pump at 1 µL min^–1^ rate while keeping the sample under stirring at 500 rpm at room temperature. Finally, the sample was diluted with 1.35 mL of milliQ water and dialysed against water (×5) using Spectra/Por 6 Dialysis Tubing, 3.5 kD MWCO (Spectrum labs). The polymer vesicle solution was further diluted to 1.5 mg mL^–1^ in PBS and imaged.

#### Ferritin

Equine ferritin was purchased from Sigma‐Aldrich and diluted at 2 mg mL^–1^ in PBS.

### Imaging methods

All the materials were imaged in solution with the Ocean holder, manufactured by *DENSsolution*. In the holder, the samples were protected from vacuum by entrapping them into a liquid chamber made by two chips of silicon nitride (Si*_x_* N*_y_*). One of the chips had a 200 nm‐spacer which allowed for the liquid sample to be channelled into the liquid cell. Both chips had a 50 nm thick electron transparent viewing window at their geometrical centre with size 10 µm × 200 µm. The spacer is designed to keep the thickness of the liquid layer constant during the experiment. However, in practical terms the thickness of the observation window is the result of the height of the spacer, that is 200 nm and the bulging effect experienced by the Si*_x_* N*_y_* windows when the holder is inserted into the microscope. The window bulging effect has been reported elsewhere (De Jonge *et al*., 2009) and is due to the he differential pressure between the microscope column and the interior of the liquid cell. The poly(methyl methacrylate) PMMA protective layer covering the Si*_x_* N*_y_* cell chips was removed by rinsing the chips in HPLC‐graded acetone and HPLC‐graded isopropanol for 5 min each. Then, the chips surface was further cleaned by air plasma discharge for 13 min which increased the surface hydrophilicity. The cleaned chips were placed into the liquid holder, where 1.5 µL of the sample was deposited onto the bottom chip and enclosed with the top chip. After sealing the liquid cell and holder, 300 µL of the liquid sample was injected with a peristaltic pump at 20 µL min^–1^ via the inlet tube until the liquid cell, and outlet tubes were filled in with the sample. Inlet and outlet tubes were then connected forming a closed loop with the liquid cell. The holder was allowed to rest for 5 min before inserting it in the microscope to avoid convection effects which may alter the Brownian motion of the nanostructures in the liquid media. The experiments were performed in static condition.

The images used to implement the proposed denoising algorithm were produced by a system comprises a JEOL JEM‐2200FS transmission electron microscope TEM equipped with a field emission gun (FEG) at 200 kV, and an in‐column Omega filter. Two imaging modes, transmission electron microscopy TEM and scanning transmission electron microscopy STEM, were used to demonstrate that the imaging process here presented works independently from the imaging modes and organic samples used. In TEM mode, the camera was the direct detection device DDD *in situ* K2‐IS camera from Gatan, which allowed low‐dose imaging modes facilitating both high spatial (3838 × 3710 pixels) and temporal resolution. Images were acquired in both counted, Figures 2 and Figure 3, and linear mode, Figure 4, with an exposure time of 0.1 s. Electron doses for the images shown in Figures 2, 3, 4 were 0.148e^−^/Å^2^, 0.517e^−^/Å^2^, 6.429e^−^/Å^2^, respectively. In STEM mode, the Hamamatsu screening camera of the microscope was used (2048 × 2048 pixels). STEM images (Figures 6A, E) were collected at a dwell time of 3 s and a total dose of 47.590e^−^/Å^2^ and 74.335e^−^/Å^2^, respectively.

### Imaging processing methods

From a mathematical point of view, a digital image can be described by using Eq. (1).

(1) $$\;I_{N}=I_{O}\times B+N,$$

Where *I_N_* is the result of the imaging process, *I_O_* models the original noiseless image, *B* the blurring function and *N* the noise component, here considered as additive. The objective of the proposed pipeline is to restore the original image $I_{O}$, suppressing the noise *N* and identifying the blurring matrix *B* (Banham & Katsaggelos, 1997). However, the high variety of sources that can produce noise makes the distribution of the noise hard to estimate (Kushwaha *et al*., 2011). For conventional TEM images the blurring term *B* does not constitute a true problem and is compensated by the contrast transfer function (CTF) estimation, which models the distortion introduced by the microscope (Rohou & Grigorieff, 2015). Unfortunately, none of the image restoration algorithms developed for conventional TEM (Narasimha *et al*., 2008) are able to account for the presence of liquid media in the imaging process. Despite the presence of the liquid that alters the *B* term, Eq. (1) can still be considered valid for LTEM images.

**Fig. 1:**
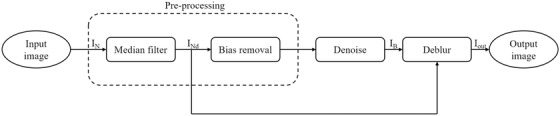
Flow chart modelling the pipeline used for restoring the original sharp and noiseless image recorded via LTEM. The image recorded by LTEM (*I_N_*) is first preprocessed by removing salt‐and‐pepper noise (*I_Nd_*) and any source of bias. Next, the resulting image is processed by the denoising algorithm to suppress the noise. The output image at this stage (*I*_B_) together with the median filtered image (*I_Nd_*) are set as input to the deblurring algorithm, which restores the original sharpness of the signal.

The approach proposed in this work is a two‐stage single input pipeline, aiming to recover the noiseless image $I_{O}$. The first stage of the pipeline is responsible for identifying and suppressing the noise, whilst the second stage restores the sharpness of the image, estimating and removing the blurring function. Figure 1 shows the flowchart of the pipeline, highlighting inputs and outputs of each stage.

**Fig. 2:**
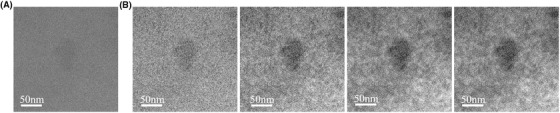
PID algorithm results when executing different number of iterations performed on PMPC‐PDPA particles when imaged via LTEM in TEM mode. (A) Original raw image as provided by the K2 camera. (B) From left to right, the outcome of the PID algorithm iterated 10, 20, 30 and 40 times respectively. The level of noise is reduced with respect to the number of iterations, but no significant differences between 30 and 40 iterations images can be noticed.

The first preprocessing block receives in input the LTEM image, which is comprised of the image data and the metadata, a text file containing the settings of the microscope. First, the image data is stored, while the metadata is discarded, as it provides no value for the image analysis. Then, the image data is processed by applying the median filter, which removes the very black and very white noise pixels corrupting the image (Chan *et al*., 2005). Additionally, the image is divided into different patches that is selected areas of pixels. The mean intensity values of the patches are then subtracted from the patches intensity in order to correct nonuniform illumination and remove any source of bias resulting from the illumination. Consequently, the intensity values of the patches are centred to 0, responding uniformly to the different steps of the pipeline (Stark, 2000). The output of the preprocessing step is fed in input to the denoising block which applies the Progressive Image Denoising (PID) algorithm (Knaus & Zwicker, 2014) in Figure 1. This method iteratively processes images in three steps: (i) separates the noise from the actual signal, (ii) estimates the noise component for every iteration and finally (iii) applies a dual‐domain image denoising (DDID) filter on both spatial and Fourier domains (Knaus & Zwicker, 2013). A great advantage of using the PID denoising algorithm relies on the absence of artefacts in the outcome of the process, as proven by Knaus & Zwicker (2014). Due to the iterative structure of the method, the most critical parameter affecting the algorithm performance is the number of iterations. Figure 2 shows an example of LTEM image of PMPC‐PDPA particles in PBS solution where the algorithm PID has been applied.

The results of the proposed pipeline method are shown when varying the number of iterations in Figure 2(B). A quantification of the noise removed from the algorithm can be performed by comparing the original noiseless image to the output of the pipeline. However, in LTEM the noiseless original image is never available, and any quantitative analysis of the goodness of the algorithm results impossible to perform (Mohammad‐Djafari, 2013).

From the results shown in Figure 2, it emerges how the noise decreases proportionally to the number of iterations. The images produced after performing 30 iterations do not significantly differ with respect to each other. Consequently, 30 iterations was set as default value in order to reduce the complexity of the algorithm.

After the noise has been suppressed, the image can enter the deblurring final stage of the algorithm (Fig. 1). Some preliminary considerations must be taken into account regarding the holder used for imaging in liquid phase. As mentioned above the holder encloses liquid samples into a liquid cell and between two electron transparent Si*_x_* N*_y_* windows. However, when the holder is inserted into the microscope, the differential pressure existing between the interior of the liquid cell and the microscope generates a bulging effect on the observation windows. The bulging experienced by the flexible silicon nitride observation windows in turn increases the thickness of the liquid layer enclosed by the liquid cell. The variation in liquid thickness however is not uniform across the liquid cell, but it seems to reach its maximum value at the middle of the observation windows. Subsequently, the range of thicknesses adopted by the liquid layer can be matched to local values of the blurring function that corrupts the image. The blurring function corrupting the LTEM images comprises the local blurring contribute of the liquid and the CTF of the microscope. Therefore, estimating *a priori* a global blurring function is impossible. The general procedure employed in the present investigation involved estimating the blurring function, from now on referred to as *kernel*, and then deconvolving it to the blurred image to restore sharpness. From a mathematical point of view, this scenario can be described as a blind deconvolution problem. In order to solve the blind deconvolution problem, we extended the methodology reported by Yuan *et al*. (2007) by splitting the image into different areas, estimating the kernel of each patch. This procedure results in a local implementation of the method proposed by Yuan. Further on, this local extension of Yuan's method will be referred to as LED (Local Extension Deblur). It is worth specifying that to further reduce the variation of the thickness of the liquid layer, the images were recorded close to the corners of the observation windows, where the thickness is at its lowest value.

Both Yuan's and LED method require two images in input: a blurred low noise image, containing information about the structure and a sharp noisy image, carrying information about the details (Yuan *et al*., 2007). We used the PID image as the blurred low noise image, and the median filtered image as the sharp noisy image. The image to set as second input was median filtered in order to improve the deblurring result by excluding the salt‐and‐pepper noise from the process, without losing the sharp structure of the image. The median filtered image produced better quality results if chosen as the initial condition in the iterative estimation process of the kernel. In order to avoid undesired copies of image features appearing in proximity of sharp discontinuities also known as `ringing' artefacts (Fergus *et al*., 2006), we performed a deconvolution step to the residual image. This latter models the difference between the original and the noisy images during the kernel estimation process (Yuan *et al*., 2007).

A GitHub repository has been created, describing the implementation of the method and providing a folder containing the Matlab scripts. The repository can be found at the following link https://github.com/GabrieleMarchello/LPEM-post-processing-pipeline.

The various denoising algorithms employed in this study were executed in a machine Ubuntu Linux 16.04 64‐bit operative system with the following hardware specifications: two central processing units (CPU) Intel Xeon Gold 5118 2.3 GHz with 12 cores each, 128 GB (8 × 16 GB) 2666 MHz DDR4 RAM, 512 GB class 20 solid state drive (SSD) and dual SLI NVIDIA Quadro P5000 16 GB as GPUs.

## Results

**Fig. 3:**
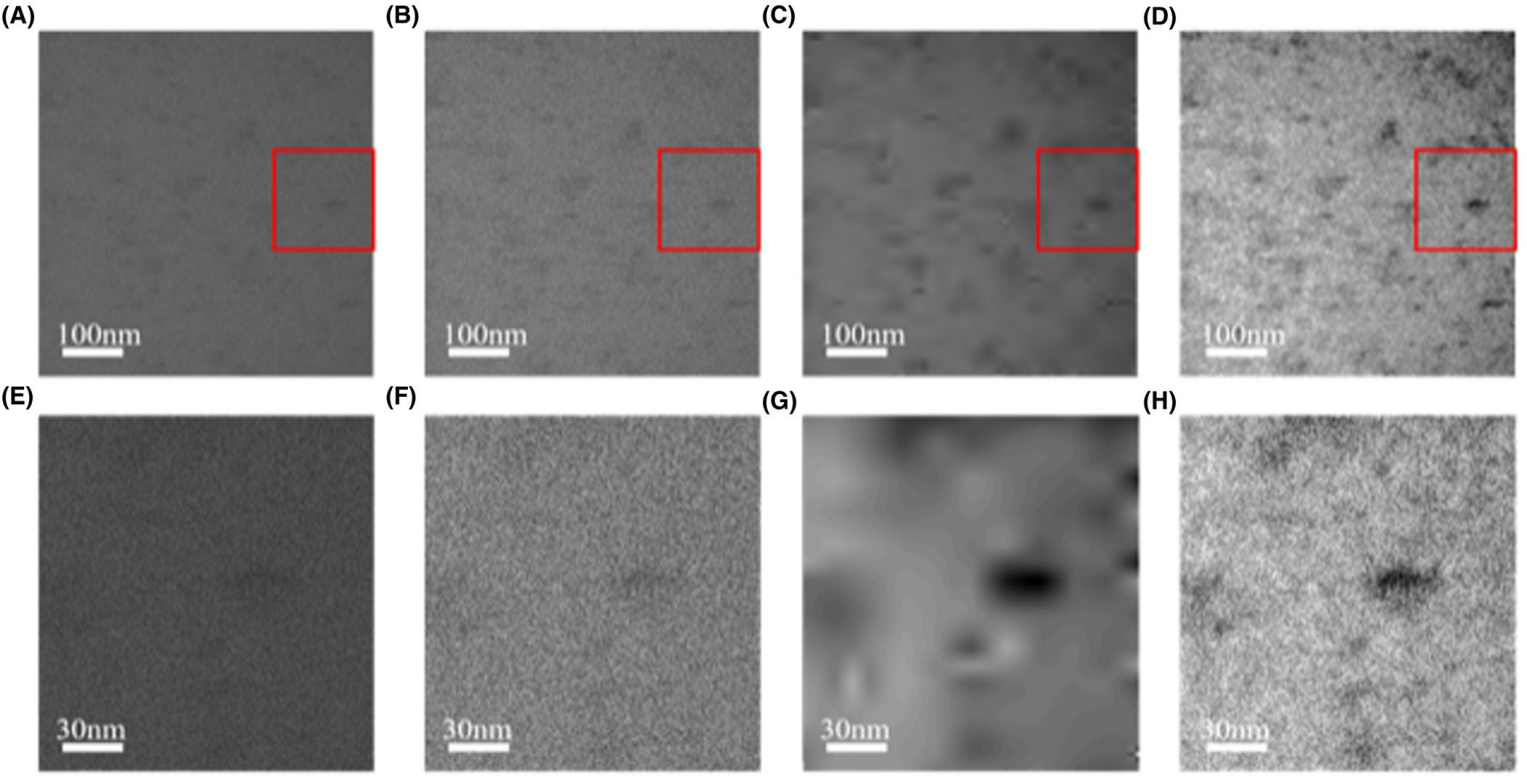
Comparison of different denoising algorithms performed on ferritin imaged via LTEM in TEM mode. (A) Original raw image of ferritin as provided by the K2 camera. (B) The outcome of the median filter, (C) wavelet denoising and (D) PID algorithm. The median filter results to be not very effective, whilst the wavelet denoising removes not only the noise, but also the actual signal, resulting in a very smoothed image. Conversely, the PID algorithm removes a significant component of the noise, preserving the features in original image. (E)–(H) The area surrounding a particle corresponding to the red box in (A)–(D), respectively.

The pipeline herein proposed extends and adapts to liquid imaging by employing two of the state‐of‐the‐art methods well established within the current image denoising and deblurring research fields. The PID algorithm was proven to ensure high performance in image denoising, as shown in Figure 3 comparing three different denoising algorithms applied to the same image of ferritin in PBS (Marchello *et al*., 2019). Figure 3(A) depicts the original noisy image, set as reference. Figures 3(B)–(D) show the outcome of the denoising processes performed via median filter, wavelet denoising process (Mallat, 2009), and the PID algorithm, respectively. The median filter and the wavelet denoising algorithm are two of the most common tools used in image analysis. However, the PID algorithm produces higher quality images (see Figs. 3B–D) and it is computationally demanding (Table 1).

**Table 1:** Reports the execution times of the various denoising algorithms employed in this study

	Median filter	Wavelet denoising	PID
Figure 2	00 h 00’ 28’’	00 h 01’ 06’’	01 h 24’ 19’’
Figure 3	00 h 00’ 31’’	00 h 00’ 56’’	01 h 31’ 27’’

**Fig. 4:**
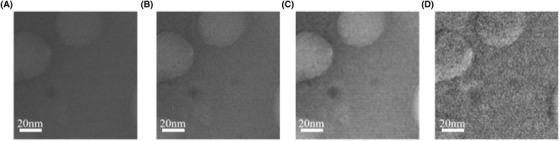
Comparison on the performances of the deblurring algorithm performed on ferritin imaged via LTEM in TEM mode. (A) Original raw image of ferritin as provided by the K2 camera. (B) PID denoised image. (C) Deblurred image produced by the original Yuan's method. (D) LED deblurred image.

The effect of the deblurring algorithm on the image of ferritin obtained via LTEM can be further appreciated in Figure 4, where the LED algorithm in Figure 4(D) was applied to the raw image in Figure 4(A) and compared with Yuan's method in Figure 4(C). The PID denoised image is shown as reference in Figure 4(B).

**Fig. 5:**
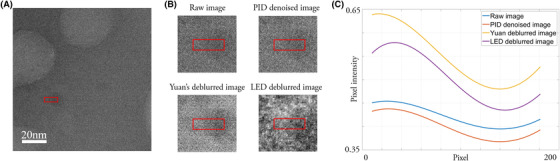
Edge analysis performed on ferritin imaged via LTEM in TEM mode. (A) Raw image as provided by the K2 showing the region of interest ROI area (red rectangle) selected near the edge. (B) Selected ROI in the four different images displayed in (C) Interpolated pixel profiles averaged along the vertical direction. The edge profile extracted from the raw, the PID denoised, Yuan's deblurred and LED deblurred images are shown in blue, red, yellow and purple respectively.

In order to understand better and evaluate the performance of both deblurring algorithms, Yuan's global and the reported LED algorithm, a region of interest (ROI) with a fixed number of pixels was chosen perpendicular to the edge of a particle of ferritin, as shown in the images displayed in Figure 4. The chosen ROI was then cropped in the four images as shown in Figure 5.

The selected ROI were flattened along the vertical direction, averaging the intensity of the pixels, in order to reduce the variation of intensity. The pixel profiles were then interpolated hence obtaining the four smoothed functions shown in Figure 5(C). The profile in blue and red, extracted from the raw and the PID denoised images respectively, result almost constant highlighting a mild variation in the pixel intensity close to the location of the edge. Conversely, Yuan's method (yellow plot in Fig. 5C) stresses the variation of the pixel intensity between the light background with high intensity values and the dark particle of ferritin with low intensity values across the edge. However, its sinusoidal function representation is wide, highlighting a distortion effect due to blur, which moves the peak of the function to the further left. On the contrary, the LED technique (purple plot in Fig. 5C) enhances the intensity values and decreases the width of the sinusoidal wave, resulting in a sharper image. To produce the results shown in Figure 5, the image was divided into areas of 128 × 128 pixels, with a stride that is the shift between consecutive areas of 8 pixels. The execution of the deblurring algorithm with these settings lasted circa 3 h. The processing time was strongly influenced by the value of the stride. In this way for smaller strides, the execution times were longer but delivered better results than for larger strides. Thus, the previous values were set as standards for further computations.

**Fig. 6:**
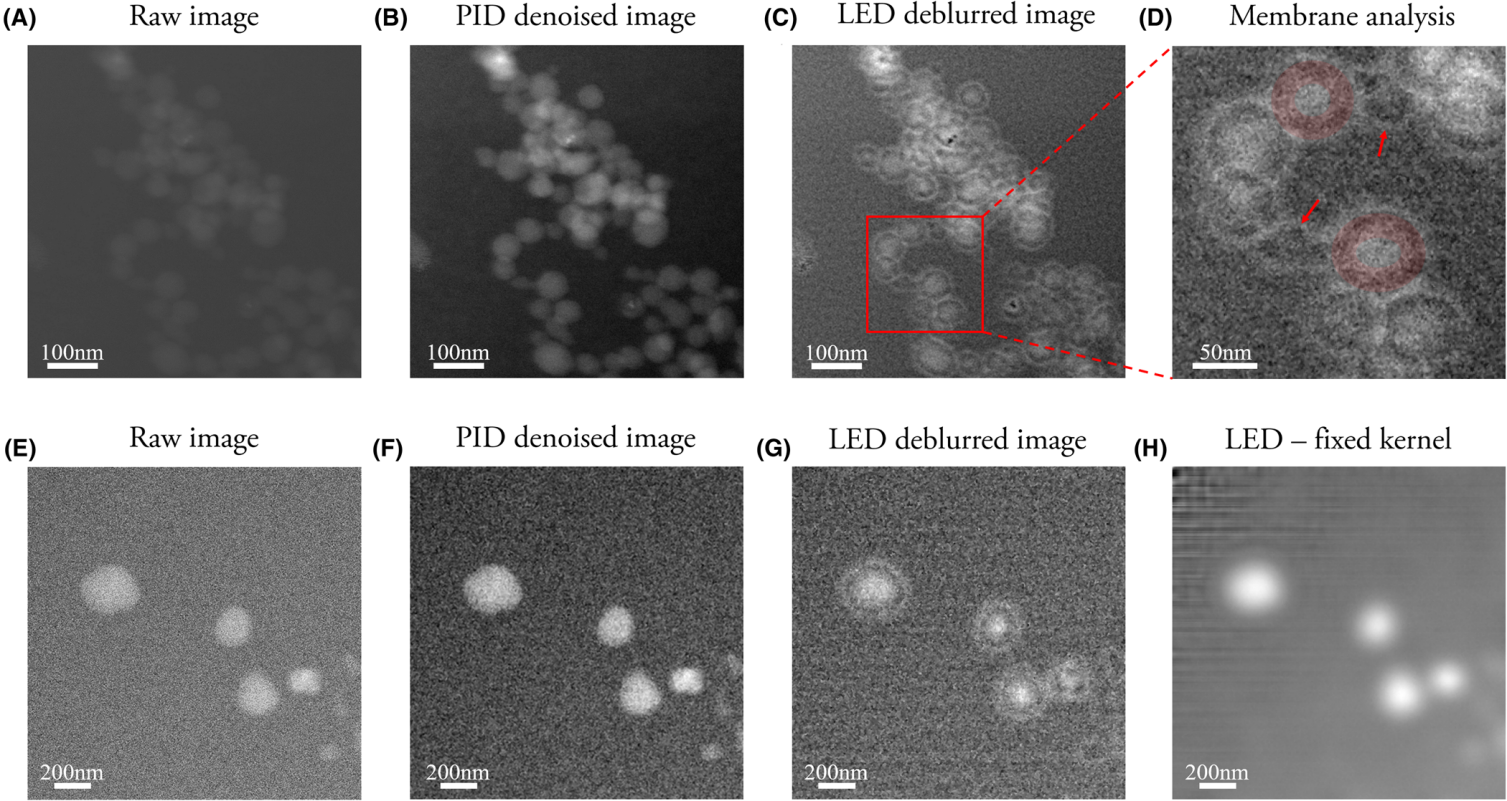
Membrane analysis of PEG‐PMET polymer nanoparticle obtained via LTEM in STEM mode dark field. (A), (E) Original raw images as provided by the microscope detector in STEM mode. (B), (F) PID filtered images obtained from (A) and (E), respectively, corresponding to the outcome of the first stage of the pipeline. (C), (G) Deblurred images, corresponding to the output of the pipeline. (D) Zoomed area of (C) highlighting how the membranes is only present in the vesicular structures and not on micelles. The red halos highlight the membranes, while the red arrows point at the micelles. (H) The kernel estimated during the deblurring process of (C) were set in input to the deblurring process of (G). The result (H) is an image with a very high level of distortion and showing no membranes.

The most significant outcome achieved by the proposed image analysis method arises in its ability to unveil details in images of organic materials obtained by LTEM, details that are otherwise hidden below noise and blur effects. An example of the output generated by the different stages of the pipeline is illustrated in Figure 6. The noisy image in Figure 6(A) depicts PEG‐PMET vesicles and micelles in solution obtained via LTEM in STEM mode. The raw images in Figures 6(A), (E) display aggregation of spherical nanoparticles, but it is not possible to discern whether the nano structures are membrane‐bound that is vesicles or solid‐core spherical structures that is micelles. The application of the full pipeline including the LED deblurring algorithm highlights the presence of the vesicle membranes and makes them physically measurable (Figs. 6C,G). To ensure that the membrane features are not artefacts introduced by the deconvolution algorithm, we run two different tests. In Figure 6, the intermediate results of the pipeline are presented. Figures 6(A), (E) show the two raw images used for the membrane analysis, Figures 6(B), (F) the corresponding PID denoised images and Figures 6(C), (G) the deblurred images. First, the image containing polymer vesicles and micelles in Figure 6(A) was processed, following the same procedure as described above. Figure 6(D) depicts a zoomed region of the deblurred Figure 6(C), containing vesicles with membranes highlighted by the red halos, and micelles where there is no presence of membranes, pointed by the two red arrows. Second, the kernels that were estimated while processing the image in Figure 6(C) were constrained as input to the deblurring process of the image in Figure 6(F). The result was shown in Figure 6(C) and it clarified how a different kernel distorts the original image, without generating any membranes. Accordingly, the membranes seen in Figure 6(G) were not thought to be artefacts produced by the deconvolution, which removed only the blur introduced by the liquid nature of the sample.

## Conclusions

The pipeline presented here proposes a novel method for restoring images of soft organic materials obtained by LTEM both in TEM and STEM modes, which are highly corrupted by noise and distorted by the liquid nature of the samples. In addition to noise and distortion effects, low exposure times and fast imaging conditions required to capture sample dynamics adds undesired effects to the LTEM imaging process. The end‐to‐end image analysis method has the ability to recover the original images together with their sharpness, without introducing any artefacts. Most notably, the deconvolution involved in the deblurring algorithm offers the great advantage of unveiling image details, which allows a better understanding of the nature, structure and ultimately the function of the investigated structures. Moreover, this fully automatised method efficiently allows to process dozens of images in few hours, without the need of any human interactions, boosting the performance of LTEM image analysis.
